# The combination of bevacizumab/Avastin and erlotinib/Tarceva is relevant for the treatment of metastatic renal cell carcinoma: the role of a synonymous mutation of the EGFR receptor

**DOI:** 10.7150/thno.38346

**Published:** 2020-01-01

**Authors:** Renaud Grépin, Mélanie Guyot, Aurore Dumond, Jérôme Durivault, Damien Ambrosetti, Jean-François Roussel, Florence Dupré, Hervé Quintens, Gilles Pagès

**Affiliations:** 1 Centre Scientifique de Monaco, Biomedical Department, 8 Quai Antoine Ier, MC-98000 Monaco, Principality of Monaco.; 2 University Cote d'Azur, Institute for research on cancer and aging of Nice (IRCAN) CNRS UMR 7284/ INSERM U 1081 3; 3 Department of Pathology, Nice University Hospital, University of Nice Sophia Antipolis.; 4 Centre Hospitalier Princesse Grace, Pathology department, Monaco.; 5 Centre Hospitalier Princesse Grace, Urology department, Monaco.

## Abstract

Metastatic clear cell renal cell carcinomas (mRCC) over-express the vascular endothelial growth factor (VEGF). Hence, the anti-VEGF antibody bevacizumab/Avastin (BVZ) combined with interferon alpha (IFN) was approved for the treatment of mRCC. However, approval was lost in July 2016 due to the absence of sustained efficacy. We previously showed that BVZ accelerates tumor growth in experimental models of mRCC in mice, results in part explained by down-regulation of the phospho tyrosine phosphatase receptor kappa (PTPRκ) in tumor cells. The epidermal growth factor receptor (EGFR) is a direct target of PTPRκ. Its down-regulation leads to constitutive activation of EGFR, an observation which prompted us to test the effect of the EGFR inhibitor erlotinib/Tarceva (ERLO) in addition to BVZ/IFN. The influence of the long non-coding RNA, EGFR-AS1, on ERLO efficacy was also addressed.

**Methods**: The effect of BVZ/IFN/ERLO was tested on the growth of experimental tumors in nude mice. The presence of germline mutation in the EGFR was evaluated on cell lines and primary RCC cells. *In vitro* translation and transfections of expression vectors coding the wild-type or the EGFR mutated gene in HEK-293 cells were used to test the role of EGFR mutation of the ERLO efficacy. Correlation between EGFR/EGFR-AS1 expression and survival was analyzed with an online available data base (TCGA).

**Results:** Tumor growth was strongly reduced by the triple combination BVZ/IFN/ERLO and linked to reduced levels of pro-angiogenic/pro-inflammatory cytokines of the ELR+CXCL family and to subsequent inhibition of vascularization, a decreased number of lymphatic vessels and polarization of macrophages towards the M1 phenotype. Cells isolated from surgical resection of human tumors presented a range of sensitivity to ERLO depending on the presence of a newly detected mutation in the EGFR and to the presence of EGFR-AS1.

**Conclusions**: Our results point-out that the BVZ/IFN/ERLO combination deserves testing for the treatment of mRCC that have a specific mutation in the EGFR.

## Introduction

Before the development of anti-angiogenic therapies (AAT), the outcome of mRCC was poor. The first treatment approved for mRCC was the humanized monoclonal antibody bevacizumab/Avastin (BVZ) in combination with the standard treatment interferon alpha (IFN), the only treatment that showed a modest efficacy 1. These drugs are aimed at asphyxiating the tumors, so they should be curative but the results of pivotal clinical trials were disappointing and gave only an increase in the time to progression and in the quality of life without a major improvement in overall survival 2, 3. The reasons for this poor efficacy depend on compensative mechanisms that allow tumor cells to escape drug-mediated cell death. Acquisition of dependence on alternative signaling pathways favoring cell proliferation and invasion has been described including the c-MET 4 and the neuropilin (NRP1/NRP2) 5, 6 pathways. Myeloid cells have also been involved in the refractoriness to AAT 7. The presence of redundant pro-angiogenic factors is also one of the causes of relapse to treatments targeting the VEGF/VEGFR pathway especially the ELR+CXCL pro-angiogenic/pro-inflammatory cytokines 8, 9. Identification of markers of response to treatment is an important challenge and may favor the discovery of new potent therapeutic targets 10, 11. The epidermal growth factor receptor (EGFR) is over-expressed in mRCC probably via EGR-1 dependent activation of its promoter 12. The hypoxia-inducible factors 1, 2 (HIF-1, 2) are constitutively active in the majority of mRCC because of frequent loss of function of the von Hippel-Lindau gene that stimulates the expression of the transforming growth factor α (TGF- α), an activator of the EGFR pathway 13. Our previous results showed that the pressure of selection exerted by BVZ induced down-regulation of the phospho tyrosine phosphatase receptor kappa (PTPRκ), a natural inhibitor of EGFR activity resulting in the acquisition of increased proliferation of tumor cells 9. These cells were driven by over-activation of EGFR as attested by the level of phosphorylation and of the subsequent activation of the ERK/MAP kinase and PI3 kinase/AKT pathways. *In vitro*, the EGFR inhibitor erlotinib/Tarceva (ERLO), which is approved for the treatment of lung cancers harboring specific mutations in EGFR, strongly inhibited proliferation of cells derived from BVZ-resistant tumors 9. These results paved the way for experiments dedicated to evaluating the relevance of combinations of ERLO/BVZ/IFN to prevent acquired resistance and to improve the current therapeutic practices. The present study highlights the molecular mechanisms associated with the efficacy of combined treatments in experimental mRCC in mice and the relevance of their use in a specific fraction of patients.

## Materials and methods

### Cell lines

The Ethics departments of the University hospital, the Cancer Centre (Centre Antoine Lacassagne), Nice, France and the Princess Grace Hospital of Monaco approved this study and participants provided their written informed consent. Cells were isolated from tumors as previously described 14. RCC4, 786-O and A498 cells were from the American Type Culture Collection and were cultured in the same defined medium.

### RNA extraction and RT-PCR

Quantitative PCR (qPCR) experiments were performed after cell passage 11. One microgram of total RNA was used for reverse transcription, using the QuantiTect Reverse Transcription kit (QIAGEN, Hilden, Germany), with blend of oligo(dT) and random primers to prime first-strand synthesis. For real-time PCR, we used the master mix plus for SYBR assay (Eurogentec, Liege, Belgium). The PCR conditions were 10 minutes at 95°C followed by 40 cycles 15 seconds at 95°C, 1 minute at 60°C. The sequences of the different couples of oligo-nucleotides are detailed in supplementary Table 1.

### Antibodies

The following antibodies were used for immuno-blotting: anti-phospho ERK 1,2 and anti-tubulin (Sigma St Louis, MO), anti-phospho S6 Kinase, total anti-EGFR/HER1 and anti-pEGFR/HER1 (Cell Signaling, Cambridge, UK) and anti ERKs (Santa Cruz Biotechnology, Santa Cruz, CA references sc 93).

### Immuno-fluorescence

Tumor sections were handled as described previously 9. Sections were incubated with anti-mouse LYVE-1 polyclonal (Ab 14817, 1:200; Abcam, Cambridge, MA, USA) or monoclonal anti-α-smooth muscle actin Sigma (αSMA A2547, 1:1000; Sigma, France), and rat monoclonal anti-mouse CD31 (clone MEC 13.3, 1:1000; BD Pharmingen, Franklin Lakes, NJ, USA) antibodies.

### Measurement of hemoglobin and cytokines

Frozen tumor tissues were homogenized using a Precellys tissue homogenizer (Bertin, Montigny-le-Bretonneux, France) in cell extraction buffer (Biosource, Villebon sur Yvette, Belgium). The intra-tumor hemoglobin content, CXCL cytokines, VEGF and VEGFC were measured as previously described 9.

### Tumor xenograft experiment

Five million 786-O or A498 cells were injected subcutaneously into the flank of 5-week-old nude (nu/nu) female mice (Janvier). The tumor volume was determined with a caliper (v ¼ L _ l2 _ 0.5). When the tumor reached 100 mm3, mice were treated twice a week with control or ERLO (50 mg/kg) or BVZ (B, 7.5 mg/kg) plus IFN (9MIU) plus or minus ERLO (50 mg/kg).

This study was carried out in strict accordance with the recommendations in the Guide for the Care and Use of Laboratory Animals. Our experiments were approved by our internal ethic committee.

### Transfection experiments

The assay was performed as already described 15 in duplicate with different amounts of pcDNA4 vector carrying the wild-type and the variant EGFR sequence (two independent preparations for each construct). At the same time, 300 ng of pGL3 luciferase expression plasmids were co-transfected as an independent control of the transfection efficiency in each well. The transfection efficiency was calculated from the luciferase counts normalized to the amount of protein. Only cells that showed the same degree of transfection efficiency (difference < 20%) were analyzed.

### Statistical analysis

Statistical analyses were two-sided and were performed using R-2.12.2 for Windows. Statistical comparisons were performed using the Student *t*-test or Wilcoxon test for quantitative data.

## Results

### ERLO exerts a strong cytostatic and cytotoxic effect that depends on the mRCC cell line and inhibits the production of pro-angiogenic cytokines

**Figure 1:**
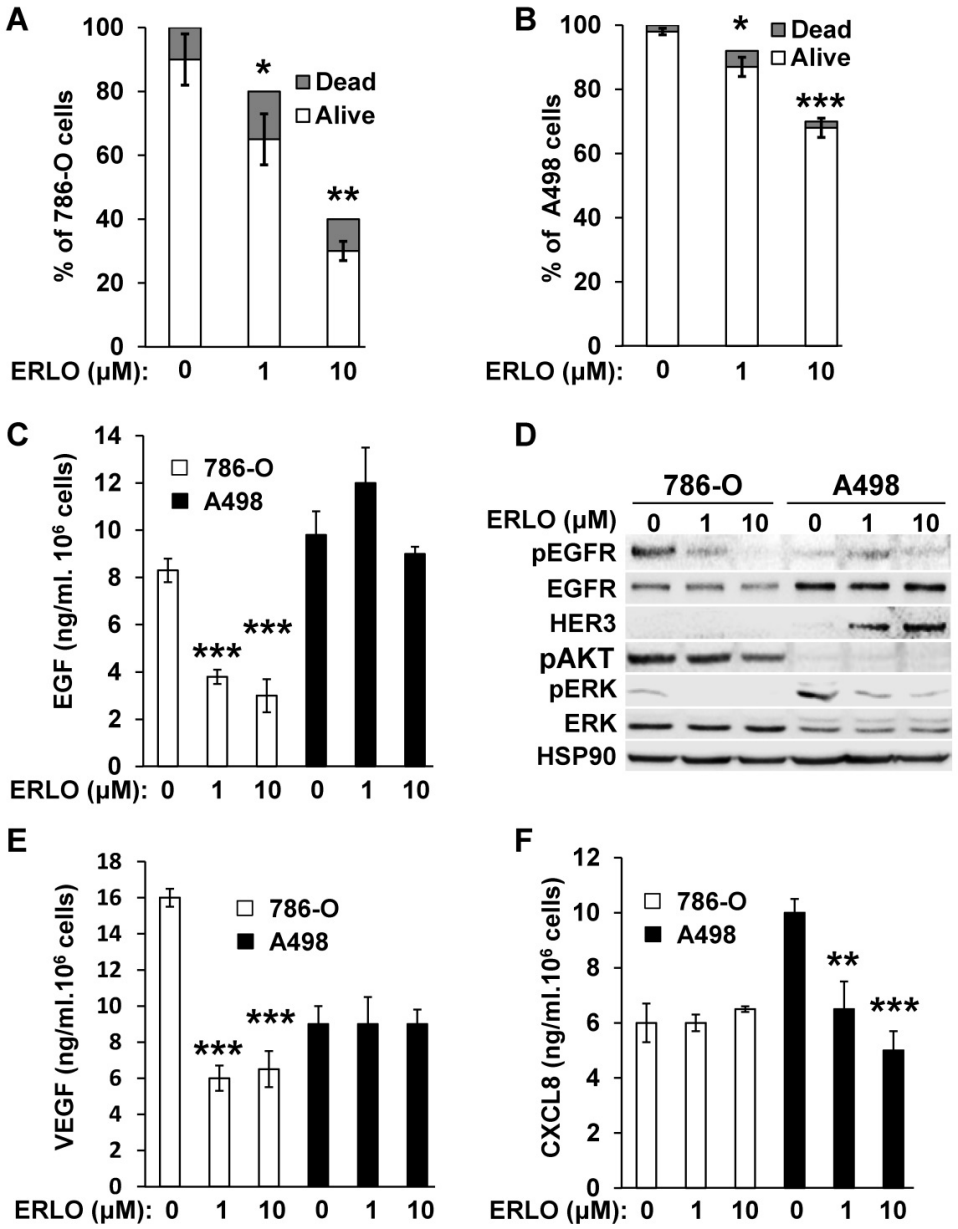
** 786-O and A498 cells present different sensitivities to ERLO**.** (A)** 786-O cells were treated with increasing concentrations of ERLO. The percentage of live and dead cells is indicated. *p<0.05; **p<0.01.** (B)** A498 cells were treated with increasing concentrations of ERLO. The percentage of live and dead cells is indicated. * p < 0.05; *** p < 0.001.** (C)** 786-O or A498 cells were treated with increasing concentrations of ERLO. EGF levels were evaluated in cell supernatants by ELISA. *** p < 0.001.** (D)** 786-O or A498 cells were treated with increasing concentrations of ERLO and were evaluated for the presence of total and active form of EGF receptor (EGFR/pEGFR), HER3, the total and active form of ERK (ERK/pERK) and the active form of AKT (pAKT) by immuno-blotting. HSP90 is shown as a loading control.** (E)** 786-O or A498 cells were treated with increasing concentrations of ERLO. VEGF levels were evaluated in cell supernatants by ELISA. *** p < 0.001.** (F)** 786-O or A498 cells were treated with increasing concentrations of ERLO. CXCL8 levels were evaluated in cell supernatants by ELISA. ** p < 0.01; *** p < 0.001.

Activation of the EGFR pathway in response to BVZ was demonstrated previously in experimental mRCC in mice 9. However, the intrinsic sensitivity to EGFR inhibitors of mRCC cells was poorly investigated. Therefore, we evaluated sensitivity using two model cell lines, 786-O and A498 cells. We obtained a dose-dependent decrease in the proliferation rate with both cell lines. The maximal reduction was of 60% and 33% for 786-O and A498 cells, respectively for the highest ERLO concentration (10 μM). Regardless of the ERLO concentration, the percentage of dead cells was equivalent (10% and 2% for 786-O and A498 cells, respectively, **Figure 1A-B**). Therefore, ERLO is cytostatic rather than cytotoxic and the cytostatic effect was stronger for 786-O cells. ERLO induced dose-dependent inhibition of EGF production by 786-O cells whereas this was not modified in A498 cells (**Figure 1C**). Therefore, the more potent effect of ERLO on cell proliferation observed for 786-O cells may be explained by inhibition of an EGF/EGFR autocrine pathway. Consistent with this, the phosphorylated/active form of EGFR (pEGFR) was dose-dependently inhibited by ERLO in 786-O cells. In A498 cells, the EGFR levels were higher compared to 786-O cells and ERLO had no incidence on pEGFR, which remained low whether or not ERLO was present, as compared to basal levels in 786-O cells (**Figure 1D** and **Figure S1**). We observed a decrease in the activity of the ERK/MAP kinase proliferation pathway for both cell lines. However, the ERK activity was lower and was more strongly inhibited by ERLO in 786-O cells. The AKT activity (pAKT) was high and was inhibited by ERLO in 786-O cells but almost undetectable in A498 cells. This result may explain the differential effect exerted by ERLO on proliferation for the two independent cell lines (**Figure 1D** and **Figure S1**).

Gefitinib, another EGFR inhibitor used to treat lung cancers 16, or cetuximab, a monoclonal antibody against EGFR, reduced the production of VEGF and CXCL8 in different cancer cells, which may explain their therapeutic efficacy 17, 18. Therefore, the effect of EGFR inhibition on secreted cytokines involved in angiogenesis was evaluated. ERLO, even at a low concentration (1 μM), inhibited VEGF production in 786-O cells but this was not modified in A498 cells (**Figure 1E**). The opposite result was observed for CXCL8 (dose-dependent inhibition in A498 cells and no effect in 786-O cells, **Figure 1F**). These results suggest that ERLO may indirectly inhibit angiogenesis through decreased production of pro-angiogenic factors by tumor cells.

### Combining BVZ/IFN with ERLO inhibited the growth of experimental mRCC in mice

**Figure 2:**
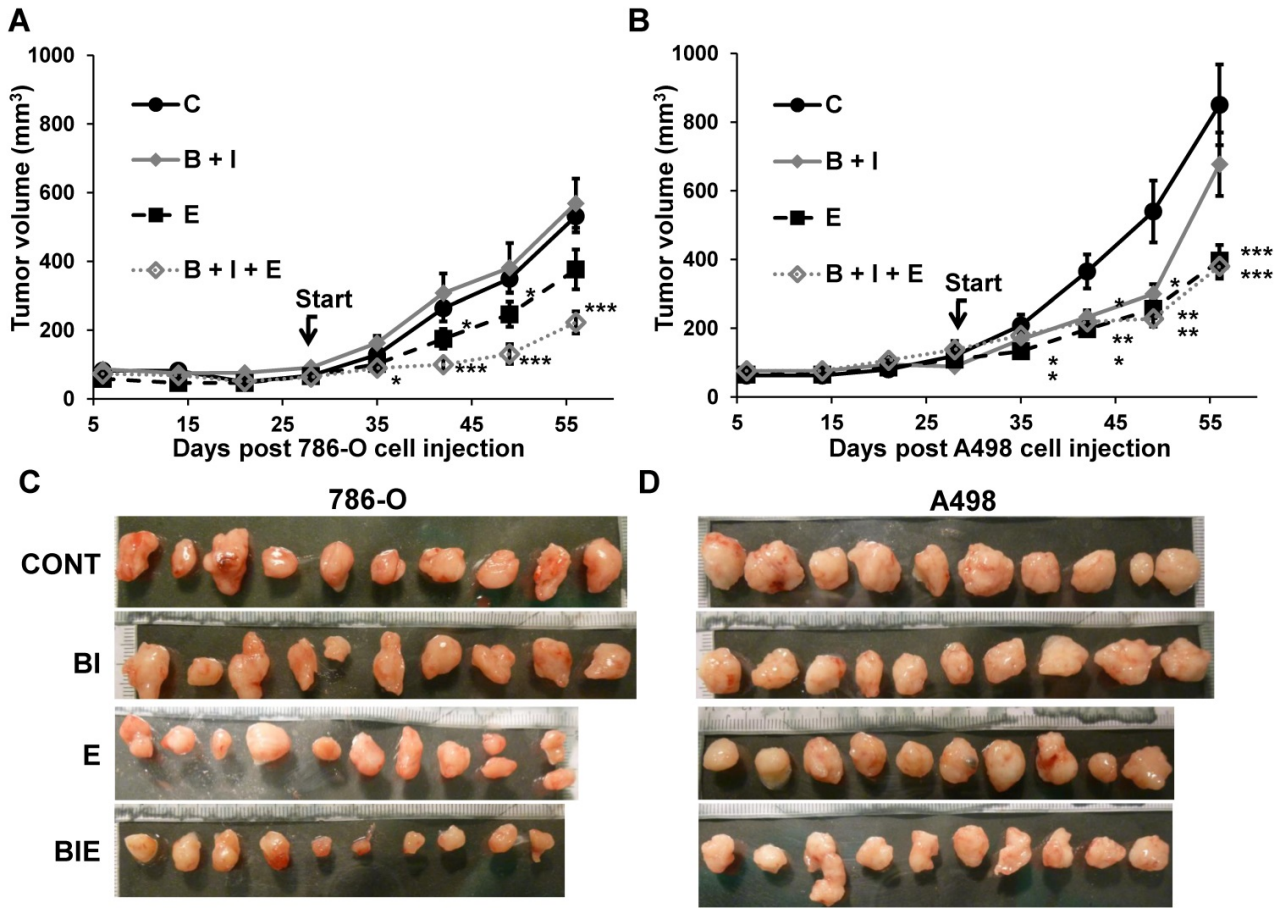
** The role of the BVZ/IFN and ERLO combination on RCC xenograft tumor growth**.** (A)** 5.10^6^ cells 786-O cells were subcutaneously injected into nude mice. Seven days after injections all mice developed tumors. 31 days after cell injection (start treatment), mice were treated twice a week with control or ERLO (E, 50 mg/kg) or BVZ (B, 7.5 mg/kg) plus IFN (I, 9MIU) plus or minus ERLO (50 mg/kg). The tumor volume is presented as the means ± s.d. (*n* = 10). Statistical differences to the untreated mice are shown: *p <0.05; *** p<0.001. **(B)** Same experiment as described in a but using A498 cells. * p < 0.05; ** p< 0.01; *** p< 0.001. * p < 0.05; *** p < 0.001. **(C)** Images of the 786-O tumors at the end of the experiments. **(D)** Images of A498 tumors at the end of the experiment.

Considering that activation of the EGFR pathway is one of the causes of relapse when on anti-angiogenic treatment with BVZ 9, we tested the effect of the combination of BVZ/IFN, one of the first approved anti-angiogenic therapies 19, with the EGFR inhibitor ERLO on the growth of two experimental mRCC tumor cell lines 786-O and A498 cells in mice. INF was used in this model to be consistent with the previously approved combination administered to the patients. Tumor growth was equivalent in the control and the BVZ/IFN groups for 786-O cells while transient inhibition was observed for A498 cells. These results reflect the intrinsic or acquired resistance observed in patients 19. ERLO alone had a modest effect on tumor growth and relapse was observed after 45 days of treatment with 786-O cells. This observation is consistent with the results of clinical trials showing the lack of anti-tumor activity associated with anti-EGFR treatments 20, 21. However, a sustained inhibitory effect was observed for A498 cells suggesting that inhibition of the EGFR pathway may hold some benefit depending on the genetic characteristics of the tumor. The triple association BVZ/IFN/ERLO was the most efficacious showing strong inhibition of tumor growth with 786-O and A498 cells although the effect of the triple combination was equivalent to ERLO alone for the latter cells (**Figure 2A-C**). These results highlight the differences in response to AAT and EGFR pathway-targeting treatments, which probably reflects tumor heterogeneity 22 or different subclasses of kidney tumors (clear cell (786-O) or papillary (A498) carcinomas 23).

### BVZ/IFN/ERLO strongly reduced tumor vessel density and prevented the development of lymphatic vessels

**Figure 3:**
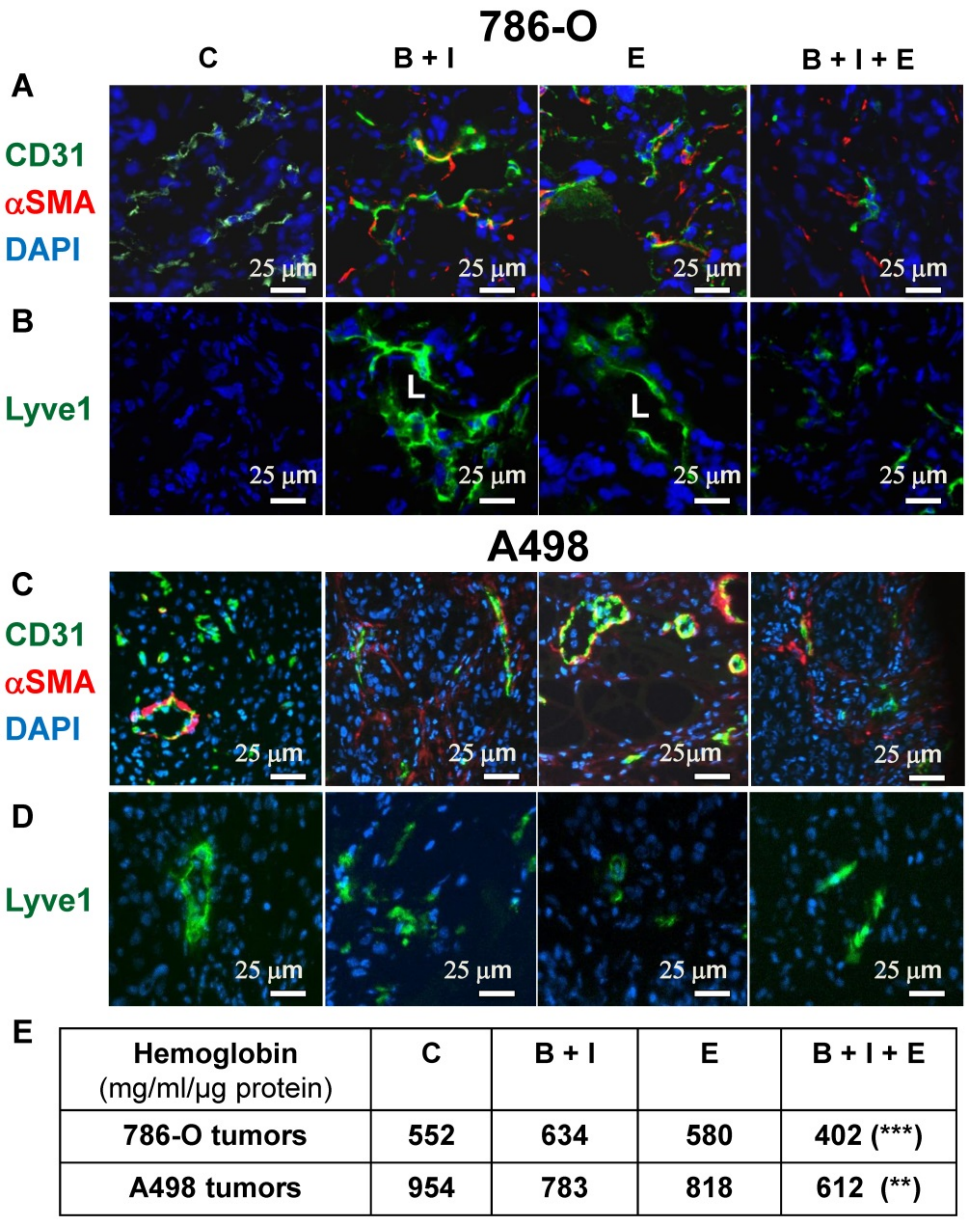
** The BVZ/IFN/ERLO combination decreased the tumor blood vessel density and prevented/inhibited the development of lymphatic vessels.** The tumor vasculature in each experimental group was detected by immuno-staining for CD31 (endothelial cells, green) and α-SMA i (pericytes, red); **(A)** 786-O cell model; **(C)** A498 cell model. LYVE-1 immuno-staining (green) shows lymphatic endothelial cells. Lymphatic vessels with lumens (L) are indicated. **(B)** 786-O model; **(D)** A498 model. Tumor sections were counterstained with 40,6-diamidino-2-phenylindole (DAPI) (nucleus, blue). **(E)** The intra-tumor amount of hemoglobin (Hg), a global read out of the blood supply, is given for both model systems and for the different experimental conditions.

We showed previously that BVZ alone stimulated experimental tumor growth. This unexpected result correlated with tumor vessel normalization and the development of a lymphatic network shown in the literature to be involved in tumor cell dissemination 9, 24. Considering these observations, we hypothesized that the triple combination may eradicate blood vessels and may prevent the development the lymphatics. The number of blood vessels decreased for 786-O tumors treated with BVZ/IFN and ERLO (**Figure 3A** and **Figure S2A**) but was not different for A498 tumors (**Figure 3C** and **Figure S2B**). However, these treatments increased the number of vessels (CD31 positive) lined with αSMA-positive cells, a pattern of vessel normalization (**Figure 3A-C** and **Figure S2A-B**). The triple combination decreased the number of blood vessels but also increased coverage with αSMA labelled cells for 786-O and A498 tumors (**Figure 3A-C** and **Figure S2A-B**). The amount of tumor hemoglobin was significantly decreased for only the triple combination suggesting that the treatment reduced tumor perfusion and/or hemorrhagic vessels (**Figure 3E**). As previously reported, BVZ stimulated the development of a lymphatic network in 786-O tumors 9. A similar result was observed when BVZ was coupled with IFN for 786-O and A498 tumors although lymphatic vessels were already present in A498 tumors in untreated mice (**Figure 3B-D** and **Figure S2A-B**). ERLO stimulated the development of lymphatics for both tumor model systems. However, the triple combination strongly reduced the BVZ/IFN- or ERLO-dependent development of the lymphatic network for both model systems (**Figure 3B-D** and **Figure S2A-B**) and the basal level of lymphatics for the A498 tumors. These results suggest that the triple combination inhibited tumor growth partly by inhibiting the formation of blood and lymphatic networks.

### Analysis of genes related to tumor angiogenesis and lymphangiogenesis, cell proliferation, immune tolerance and polarization of macrophages

To understand the better efficacy of BVZ/IFN/ERLO, we investigated the genes involved in the adaptation of cancer cells (proliferation genes) and cells of the tumor environment (immune tolerance, macrophages, pro/anti-angiogenic genes) to a given treatment. **Table 1** summarizes the modifications to the mRNA analyzed by qPCR or proteins analyzed by ELISA. First, gene expression differed for the two cell lines highlighting the importance of the tumor genetic background. However, some genes were consistently modified by the different treatments in both cell lines. PTPRκ mRNA levels were decreased by BVZ 9, but were up-regulated by BVZ/IFN in 786-O and A498 cells. Strikingly, inhibition of EGFR by ERLO induced PTPRκ only in A498 tumors. However, PTPRκ levels were decreased by the triple combination. These results suggest that the association of IFN with BVZ prevented compensatory activation of proliferation pathways mediated by a decrease in PTPRκ. However, concomitant inhibition of the VEGF and EGFR pathways resulted in down-regulation of PTPRκ. Human EGFR levels were increased by BVZ/IFN in both cell lines indicating that the compensatory mechanisms linked to VEGF/VEGFR inhibition involved the EGFR pathway. Induction of EGFR in cells of the microenvironment was also observed in response to ERLO with both cell lines indicating that EGFR inhibition was compensated by over-expression of the receptor. In both cell lines, the inhibition of the EGFR pathway was also compensated by over-expression of EGF by tumor cells only for the triple combination. The colony stimulating factor 1 and its receptor (CSF1/CSF1R) were then investigated since CSF1R is highly expressed in RCC cells because of chromosome 5q22qter amplification 25, 26. The triple combination inhibited CSF1R expression in both cell lines suggesting that the treatment indirectly targeted an autocrine proliferation pathway. Our previous observation showed that BVZ had no effect on expression of its target VEGF produced either by tumor cells or cells of the microenvironment 9. Unfortunately, the triple combination stimulated VEGF expression by tumor cells in both model systems. Moreover, VEGFC, a key player involved in metastatic dissemination via the lymphatics, was enhanced by the triple combination in both model systems. Increased VEGFC expression was consistent with the presence of lymphatic vessels observed in **Figure 2B-D**. The expression of angiogenic factors redundant for VEGF was suspected to promote BVZ resistance 9. The CXCL family of cytokines was investigated because of its involvement in RCC aggressiveness, as we previously shown 8, 9. The CXCL family of cytokines is divided into pro- and anti-angiogenic members. Only CXCL5 and CXCL7, two pro-angiogenic members, are consistently down- and up-regulated in both cell lines, respectively by the triple combination. The inflammatory context is a key player in adaptation to treatment. CD45 a tumor-infiltrating leukocyte gene was increased in 786-O tumors treated with BVZ/INF. F4/80 macrophage gene was also up-regulated by BVZ/INF or BVZ/INF /ERLO for the 786-O model and down-regulated for the triple combination in A498 tumors. The polarization of macrophages is particularly important for treatment adaptation 27. Only the triple combination consistently down-regulated expression markers of M2 macrophages (arginase and CD206) in the two tumor models. Finally, immune tolerance was investigated because of the efficacy of anti-programmed death ligand (PDL1) antibody treatment, especially for the most aggressive tumors 28. PDL1 was only detected in 786-O cells and BVZ/IFN and BVZ/IFN/ERLO strongly induced its expression. This finding is in agreement with the clinical activity of the BVZ plus atezolizumab (anti-PDL1) combo 29. According to these differences, we attempted to quantify the good and bad prognostic markers. We gave a score of 1 for a good prognostic marker, a score of -1 for a bad marker and 0 for unchanged or undetected markers. The best score (3) was obtained with BVZ/IFN treatment whereas the worst score (-3) was assigned to ERLO treatment of 786-O cells. For the A498 cells BVZ/IFN or ERLO generated the best scores. Surprisingly, triple treatment did not give the best score although tumor growth was strongly impaired. These results suggest that the triple association may select tumor cells with a more aggressive phenotype that are kept in check by the drugs.

### Cells derived from mice tumors treated with BVZ/IFN/ERLO are still sensitive to ERLO

**Figure 4:**
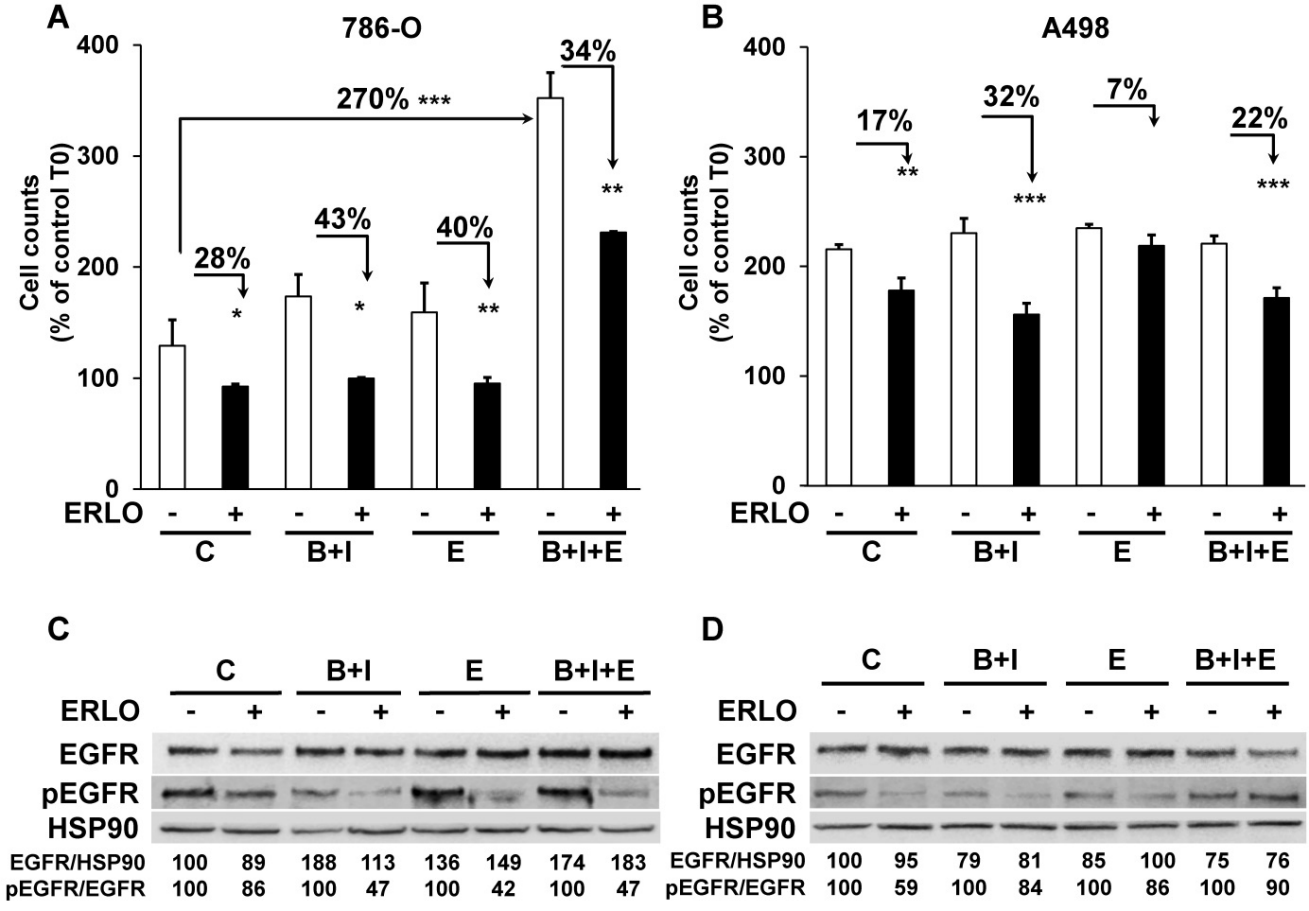
** The capacity to proliferate and the sensitivity to ERLO of cells from experimental tumors. (A)** The capacity to proliferate of 786-O cells isolated from three independent tumors from each group was tested using the MTT assay (C cells from untreated mice; B+I; cells from BVZ/IFN-treated mice; B+I+E; cells from BVZ/IFN/ERLO-treated mice) in the absence (-) or presence (+) of ERLO. **(B)** The proliferative capacity of A498 cells isolated from three independent tumors for each group in the absence (-) or presence of ERLO was tested using MTT assays. For both cell types, results are presented as the mean fold increase ± s.d. Statistical differences in the fold increase of tumor cells isolated from control mice were taken as reference values. * p < 0.05; ** p < 0.01; *** p < 0.001.** (C)** Representative 786-O cells from the four experimental groups were tested for the presence of the total and active form of EGFR (EGFR/pEGFR) in the absence (-) or presence (+) of ERLO (10 µM). HSP90 is shown as a loading control. Quantification of the relative level of EGFR (EGFR/HSP90) and pEGFR (pEGFR/EGFR) is shown. The reference values (100%) correspond to the levels of EGFR and pEGFR in cells of tumors derived from untreated mice in the absence of ERLO.** (D)** Equivalent experiments as described in **c** for the A498 model.

The different treatments generated a wide range of profiles of tumor growth. Therefore, we hypothesized that due to the selection pressure exerted by the different drugs, tumor cells acquired specific genotypic/phenotypic profiles. Thus, we analyzed their proliferation after amplification and selection from the tumors, as previously described 9. The proliferation rates forty-eight hours after seeding of cells from control, BVZ/IFN and ERLO 786-O treated-tumors were low or similar (125, 175 and 160 %, respectively, **Figure 4A**).

However, cells from BVZ/IFN/ERLO 786-O treated-tumors proliferated three times more than those from control tumors (350 %, **Figure 4A**), which reflected their strong level of EGF production (**Table 1**). The proliferation rates of A498 cells extracted from the different tumors were higher than that of 786-O cells (200 %) whereas they were lower for parental cells (**Figure S4**). However, they were similar whatever the treatment (**Figure 4B**). In these cells, the intra-tumor levels of human/mouse EGF and EGFR varied according to the treatment (**Table 1**). We showed previously that exposure to BVZ sensitized resistant cells to ERLO because of PTPRκ down-regulation 9. Consistently, 786-O and A498 cells from BVZ/IFN tumors were more sensitive to ERLO than cells from control tumors (28 % versus 43 % inhibition for 786-O cells and 17 % versus 32 % for A498 cells). This result is also consistent with increased expression of EGFR in both model systems. 786-O cells from ERLO tumors were still highly sensitive to ERLO (40 % inhibition) whereas A498/ERLO cells became insensitive (only 7 % inhibition). This result is consistent with increased expression of EGF in 786-O cells and its down-regulation in A498 cells (**Table 1**). Cells from triple-treated tumors were still sensitive to ERLO whatever the model. This persistent response to ERLO was linked to increased expression of EGF in both model systems (**Table 1** and **Figure 4A-B**). Hence, the chronic inhibition of the EGF/EGFR proliferation pathway is consistent with the *in vivo* efficacy of the triple combination.

We then analyzed the level and activity of EGFR and the sensitivity to ERLO of signaling pathways involved in cell proliferation (ERK/MAP Kinase and PI3Kinase/AKT). Total EGFR levels were increased following treatment of the 786-O model system and were slightly decreased in the A498 model. Basal levels of the phosphorylated/active form of EGFR (pEGFR) decreased in 786-O and A498 cells after BVZ/IFN treatment. This result is consistent with increased levels of PTPRκ (**Table 1** and **Figure 4C-D**). However, the decreased level of PTPRκ in cells from the triple-treated tumors resulted in a modest increase in basal pEGFR levels for both systems. ERLO inhibited pEGFR in the different cells for both cellular models except for A498 cells from the triple-treated tumors. This result reflects an alternative mechanism of EGFR activation probably through the increased expression of EGF by cells of the microenvironment (**Table 1**). Inhibition of the EGFR activity correlated with inhibition of ERK and preferentially with the AKT activity (**Figure S3A-B**). However, the persistence of ERK and AKT activity independently of the EGFR activity reflects activation of alternative proliferation pathways independent of the EGF/EGFR pathway after chronic exposure to treatments.

### Primary cells present a different sensitivity to ERLO

We showed previously that treatment response to AAT, especially to the current reference treatment sunitinib, was equivalent in metastatic patients and in primary cells derived from the patients' surgically removed tumor 14. In equivalent experiment BVZ had only a modest effect on tumor cell *in vitro*. The sensitivity to ERLO can be assessed on primary cells as well to propose this alternative treatment in case of resistance to sunitinib. The half-maximal inhibitory concentration (IC50) for ERLO and for sunitinib, is reported in **Table 2** for our reference 786-O and A498 cell lines and the already described primary cells 14. Three primary cell cultures were derived from metastatic tumors (CC, M, TF). Some cells were sensitive to both treatments (sunitinib, ERLO; 786-O, CC), to only sunitinib (A498, M) or to none of these treatments (TF). Only one primary culture (CC) was more sensitive to ERLO compared to 786-O cells (IC50 1.65 lower). M and TF cells presented a 2.2 and a 2.3-fold higher IC50 for ERLO compared to 786-O cells. To explain the relative sensitivity to ERLO of the primary cultures, we compared their relative amount of EGFR to that of our reference cell lines 786-O and A498. We also added an additional cell line obtained from the ATCC, RCC4 cells. A498 cells expressed the highest amounts of mRNA and protein (**Figure S5A-C**). EGFR mRNA levels in 786-O cells are 50% and 25% percent those of RCC4 and A498 cells respectively. However, EGFR protein levels in 786-O cells are 20% and 6.6% percent those of RCC4 and A498 cells respectively. Of note ERLO did not influence the EGFR level (**Figure S5B-C**). This discrepancy for 786-O cells may be related to the high levels of a long non-coding EGFR antisense mRNA (EGFR-AS1) already described as a marker of poor prognosis in RCC 30 and which modulates ERLO efficacy in head and neck carcinoma 31. EGFR-AS1 mRNA levels were the highest and EGFR mRNA levels were the lowest in 786-O cells (**Figure S5D**). The relationship between EGFR/EGFR-AS1 levels and tumor aggressiveness was evaluated by using the online available data of the TCGA. EGFR is overexpressed in RCC from non- metastatic (M0) and metastatic (M1) patients as compared to healthy tissue. Surprisingly, EGFR levels decreased in tumors from metastatic patients (compared M0 to M1) (**Figure S6A**). Over-expression of EGFR was indicative of a longer overall survival (OS) for M0 patients (p = 0.00209) whereas an inversion of this trend was observed for M1 patients although it did not reach statistical significance (p = 0.107, **Figure S6B-C**). In M1 patients, overexpression of EGFR was correlated to a shorter progression-free survival (PFS, p = 0.0241) and a trend was observed for a shorter disease-free survival (DFS, p = 0.0609) (**Figure S6D-E**). EGFR-AS1 is also overexpressed in RCC from M0 and M1 patients as compared to healthy tissues. No statistically significant difference was observed between M0 or M1 tumors (**Figure S7A**). High EGFR-AS1 levels were correlated with a shorter OS in M1 patients (p = 0.0468) and a trend was observed in M0 patients although non-significant (p = 0.121) (**Figure S7B-C**). However, high EGFR-AS1 levels were associated with a longer DFS in M0 patients (p = 0.0145) and a shorter PFS in M1 patients (p = 0.0434) (**Figure S7D-E**). Hence, in M0 patients a mirror image of the role of EGFR and EGFR-AS1 on OS and DFS was observed with an unexpected beneficial role of EGFR on OS. However, EGFR and EGFR-AS1 were systematically associated with shorter OS and DFS in M1 patients. These results are consistent with the pejorative role of EGFR and the relevance of its inhibition in metastatic patients.

### A silent mutation of EGFR correlated with EGFR levels and ERLO sensitivity

**Figure 5:**
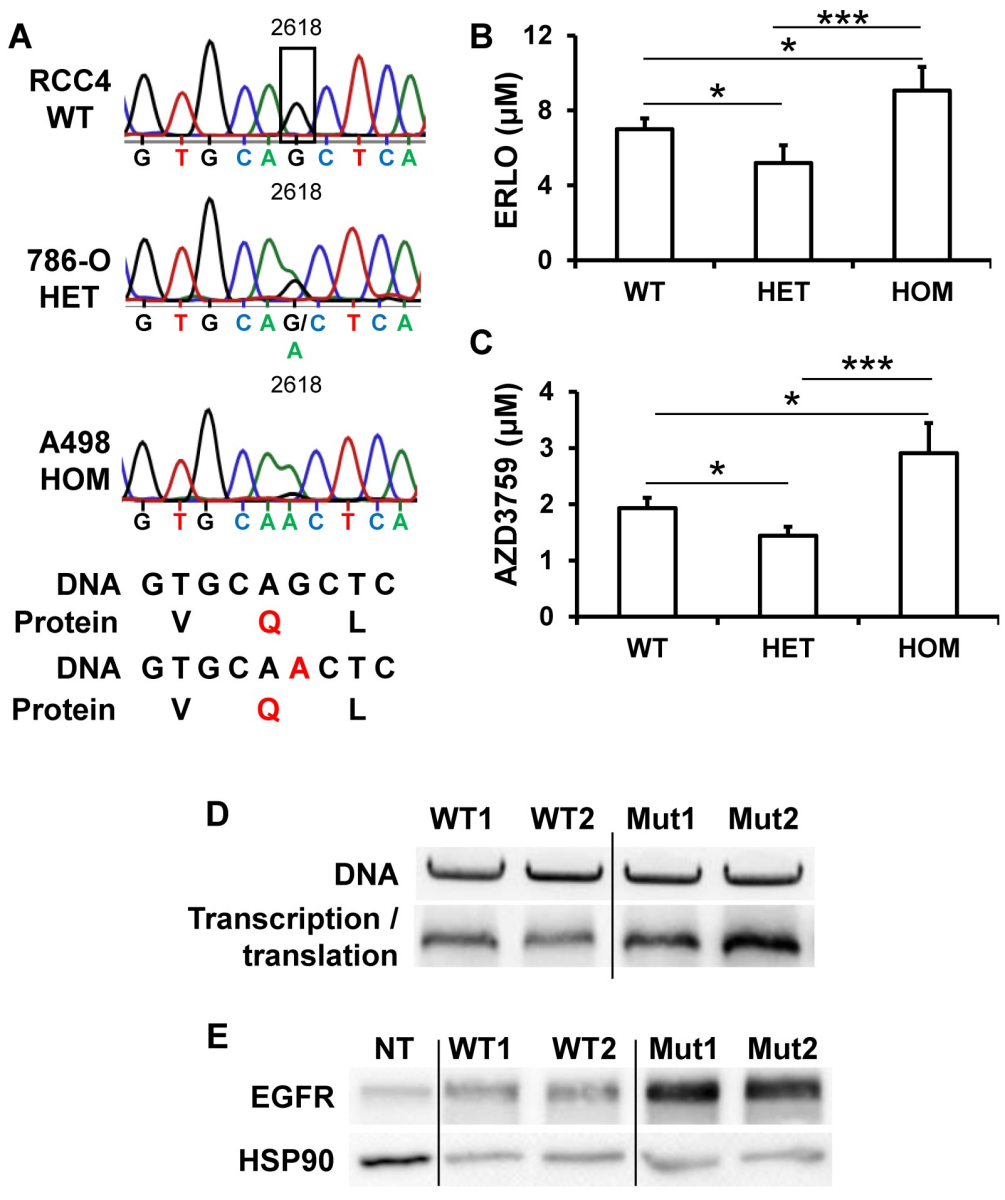
** The presence of a silent mutation in the kinase domain of EGFR is indicative of ERLO efficacy**. **(A)** Sequence chromatogram analysis of the EGFR coding region of genomic DNA obtained from RCC4, 786-O and A498 cells. **(B)** The IC50 for ERLO of the different primary cells wild-type (WT) heterozygous (HET) or homozygous (HOM) for the G 2618 A mutation was tested by MTT assays. * p < 0.05; *** p < 0.001. **(C)** Equivalent experiments as described in **(B)** for AZD3759 compound. **(D)** *In vitro* transcription and translation of two independent wild-type (WT1, WT2) and mutated (Mut1, Mut2) EGFR expression plasmids. Upper panel: equal amounts of DNA were used for *in vitro* reactions, and the quality of the plasmids was verified on agarose gels colored with ethidium bromide. Lower panel: proteins resulting from the *in vitro* transcription/translation reaction were analyzed by immuno-blotting. **(E)** 200 ng of two independent expression vectors carrying wild-type (WT1, WT2) and mutated (Mut1, Mut2) EGFR expression plasmids were transfected into HEK293 and total protein lysates were analyzed by immune-blotting. Comparison between samples was performed after the calculation of the transfection efficiency. HSP90 is shown as a loading control.

EGFR levels and its activity varied from tumor to tumor, a situation that may explain the general failure to ERLO in clinical trials. In lung cancers, for which ERLO is routinely used, EGFR protein levels and activity, that are crucial for ERLO efficacy, are never assessed before ERLO treatment. Moreover, ERLO is efficient for lung tumors but only if EGFR has mutations in the kinase domain 16, 32. To determine whether specific mutation(s) may explain the relative expression and the difference in sensitivity to ERLO of cell lines and primary cultures, we performed exome sequencing of the *EGFR* gene. The different mutations/deletions determining ERLO sensitivity in lung cancers were not detected in RCC cells 16, 32. We detected a single-nucleotide polymorphism (SNP) that modifies the codon corresponding to glutamine from CAG to a CAA (NM_005228; G 2618 to A, rs1050171), a mutation described in osteosarcoma 33 and in head and neck tumors 31, 34. RCC4 cells are wild-type (CAG codon) on both alleles, 786-O cells are heterozygous and A498 cells are homozygous for the mutation (CAA on both alleles, **Figure 5A**). The corresponding amino acid is located within the kinase domain (Q 787). This specific mutation modifies a frequently used codon for Q to a rare codon (CAG, frequent codon for Q, 73% to rare codon CAA (27%)). In addition to the differences in mRNA levels, this result may explain the difference in the total amounts of EGFR detected in the different cell lines and their sensitivity to ERLO (Supplementary Fig. S5). We then derived primary cultures from additional surgically removed tumors. 3 out of 31 primary cells (9.7%) were wild-type, 13 out of 31 (41.9%) were heterozygous and 15 out of 31 (48.4%) were homozygous for the silent mutation. We also derived primary cultures from the normal renal tissue for the corresponding patients. Normal cells were carrying the mutation suggesting its presence in the germinal state. This result was consistent with the allele distribution of this SNP in the European population (https://www.ncbi.nlm.nih.gov/SNP/snp_ref.cgi?rs=1050171). Sensitivity to ERLO was tested in the different primary cells. The IC50 for ERLO was the lowest for cells with the heterozygous mutation and the highest for the cells with the homozygous mutation and intermediate for wild-type cells (**Figure 5B**). The differences in ERLO sensitivity were confirmed using another specific EGFR inhibitor: AZD3759 (**Figure 5C**). Considering these results, we investigated whether the G2618A mutation could be responsible for the discrepancy between the mRNA and protein levels observed in the different cell lines. We hypothesized that a higher efficiency of translation of mRNA carrying the A mutation occurs. To functionally test this hypothesis, we performed an *in vitro* transcription and translation assay using an EGFR construct for both the wild-type and the mutated allele. The wild-type construct was translated less efficiently than the mutant (**Figure 5D**). To confirm these results, HEK293 cells expressing very low EGFR levels were transfected with expression vectors coding for the wild-type or the mutated EGFR. A luciferase construct was co-transfected as a control for transfection efficiency as already described 15. Comparing only samples with the same transfection efficiency, we found that the wild-type EGFR plasmid produced a lower amount of protein (**Figure 5E**). These results strongly suggest that patients carrying an homozygous wild-type genotype express the highest levels of EGFR.

## Discussion

The presence of high amounts of EGFR in mRCC cells suggested that EGFR inhibitors may have a potent therapeutic effect. A phase II clinical trial with the EGFR pharmacological inhibitor 21 and a phase I/II clinical trial using EGFR-directed antibodies gave disappointing results 35 on RCC, but the BVZ/ERLO combination appeared promising for hereditary renal cell cancer and sporadic papillary renal cell carcinoma (clinical trial NCT01130519 36). Both clinical trials on RCC did not associate EGFR inhibitors with the previously FDA-approved combination of BVZ and IFN. While remaining cautious, the differences between the results of the clinical trials and our preclinical models suggest that IFN enhances the therapeutic effect of BVZ and ERLO. The recent development of immune checkpoint inhibitors for kidney cancer strongly suggests that IFN, the first generation of immuno-therapies, is a key player for combined treatment and should be associated with anti-EGFR inhibitors for a maximal effect.

To gain insight into the related molecular mechanisms, we scrutinized the different pathways that were involved in relapse on treatment with BVZ in our previous study 9: modification to the network of blood and lymphatic vessels, compensation by redundant angiogenic factors, selection of more aggressive tumor cells and adaptation to the microenvironment. Our previous study highlighted the strong impact of BVZ on the normalization of the vascular network and the development of a VEGFC-dependent lymphatic network. In the present study, a striking difference between ERLO and BVZ/IFN treatments, alone or in combination was observed for both networks. Whereas single treatment normalized the blood vessels and stimulated the development of a lymphatic network, the triple combination was associated with a decrease in the number of blood vessels, an increase in α-SMA labelled cells and the presence of fewer or equivalent numbers of Lyve-1 positive cells. Despite the stabilization of tumor growth, the presence of lymphatic vessels 37 and α-SMA-labelled tumor associated fibroblasts 38, 39 were described as indicative of further tumor evolution. The pressure of selection mediated by the treatment, stimulated VEGFC expression by human tumor cells. Such differences are implicated in mechanisms of resistance 40. VEGFC-dependent induction by a treatment may also serve to define the best concentration of a drug that avoids such compensatory mechanisms.

Our current study was based on the BVZ-mediated decrease of PTPRκ, down-regulator of EGFR activity. However, BVZ/IFN increased PTPRκ levels. Hence, IFN indirectly decrease the activity of EGFR and other tyrosine kinase receptors that are PTPRκ targets (PDGFR, cMET, insulin receptor). EGFR is not only expressed by tumor cells but also by endothelial cells and the EGF/EGFR pathway participates in processes of tumor vascularization 41. Induction of human or mouse EGF and/or EGFR with single treatment with ERLO or BVZ/IFN may explain the increase in the number of mature blood vessels.

The decrease of CSF1R amounts with the triple treatment argues strongly for a reduction in tumor growth since the CSF1/CSF1R pathway exerts an autocrine proliferation loop in RCC and CSF1R is indicative of poor prognosis 42. Moreover, the EGF produced by tumor cells stimulated the secretion of CSF1 by cells of the microenvironment, which amplified proliferation of tumor cells 42. Any decrease in EGF or CSF1 will prevent tumor growth, a situation encountered with only the triple combination.

Triple treatment also played a prominent role on the polarization of macrophages that can alternate between pro-inflammatory (M1) and pro-tumorigenic (M2) phenotypes 43. Whereas M1 markers were not affected, M2 markers were down-regulated with BVZ/IFN/ERLO for the 786-O and A498 cellular models. M2 macrophages have been implicated in increased angiogenesis 44. Hence, down-regulation of M2 macrophages may explain the decrease in micro-vessel density in tumors with BVZ/IFN/ERLO. The M2 phenotype is stimulated by the CSF1 pathway 45, which is consistent with the upregulation of M2 markers in the presence of ERLO.

The prognostic score we generated for the different combinations, was not indicative of the ideal treatment and differed for the two tumor models. These results suggest that these treatments may be efficient but need to be used with caution depending on specific genetic characteristics. Cells isolated from tumors exposed to triple treatment showed a higher ability to proliferate in only one model. However, the cells were still sensitive to ERLO in both models. This result suggests that ERLO must be maintained to prevent acceleration of tumor growth.

The increase in PDL1, which participates in evasion of immune surveillance 46, is not in favor of the use of the triple combination. Since treatments targeting the PD-1/PDL1 axis have been approved for the treatment of mRCC 47, it may be used at relapse when on the triple combination. However, despite expression of PDL1 by tumor cells, the presence of IFN may still induce proliferation of cytotoxic T lymphocytes and may maintain immune surveillance.

EGFR inhibitors are currently used for the treatment of lung cancers, but treatment is efficient only if the receptor has specific mutations in the kinase domain 16, 32. Moreover, a mutation that antagonizes the efficacy of the major EGFR inhibitor ERLO was recently discovered 48. Although the presence of these mutations depends on the cancer types, they are very rare in mRCC 49. A specific mutation of the kinase domain of EGFR was recently described in mRCC but in another position than that described in the literature 50. The discovery of a specific mutation in EGFR in mRCC may constitute a predictive marker of sensitivity/resistance to EGFR inhibitors to increase the treatment arsenal in case of therapeutic impasse. However, we were troubled by the differences between *in vivo* and *in vitro* results (better efficacy of ERLO for the A498 model *in vivo* and better efficacy of ERLO for the 786-O model *in vitro*). This discrepancy may be explained by the ability of the different tumor cells to shape the microenvironment. As illustrated in Table 1, human and mouse EGFR is induced by ERLO in the A498 model but only mouse EGFR is induced in the 786-O model. Human EGF is induced in the 786-O model and mouse EGF is induced in the A498 model. Hence, it is reasonable to think that the growth of A498 tumors is more addicted to the EGF/EGFR pathway and therefore more sensitive to ERLO.

Another possibility is the difference in perfusion (measurement of hemoglobin levels) of the 786-O versus the A498 model. Strikingly, the hemoglobin in A498 tumors is twice that of 786-O tumors. Therefore, ERLO may have a better access to tumor cells in the A498 model.

Finally, the EGFR genotype status is unknown in nude mice and may mitigate the relative efficacy of ERLO. Nevertheless, these experiments aimed at demonstrating the relevance of adding EGFR inhibitors to the previously approved BVZ/IFN treatment. Since the mutation appears as germinal, we can estimate that the triple combination would be more efficient for patients with a heterozygous genotype.

The analysis of genome sequences in cancer revealed that silent mutations can control the speed of mRNA translation, mRNA folding, pre-mRNA splicing, and through translational pausing, the folding of proteins 51. Moreover, mRNA containing CAG codons are less translated than those with the CAA codon. Hence, silent mutations are driver mutations for tumor development and constitute predictive markers of resistance to a given treatment 15. The G2618A mutation modifies a frequently used codon for Q to a rare codon. Its presence in the germinal state suggests that the patients with kidney cancers carrying a homozygous mutation (A/A) are intrinsically resistant to EGFR inhibitors. However, the opposite situation was observed for patients with head and neck cancers (higher sensitivity to ERLO if A/A), a phenotype depending on the degradation of the long non-coding RNA EGFR-AS1 31. The A/A genotype destabilizes the EGFR-AS1 resulting in EGFR inhibitors sensitivity in head and neck tumors. On the contrary, EGFR-AS1 levels are the lowest in homozygous wild-type (G/G) RCC cells. Strikingly, EGFR-AS1 expression is very low and it is not correlated to survival in head and neck tumors (TCGA analysis), which is exactly the contrary in RCC. The regulation of protein expression in heterozygous cells remains unclear and was a not addressed in the seminal paper of Tan and colleagues on head and neck cancers 31. The presence of the mutation (A/A) creates a high affinity binding site for miR219. Such an interaction may also lower mRNA translation in heterozygous RCC cells and increased sensitivity to EGFR inhibitors. Hence, the A/A mutation and the presence of miR219 may serve as a rheostat for down-regulating EGFR levels. This mechanism is consistent with the tumor suppressor role of miR219 52. Surprisingly, EGFR-AS1 was recently described as an indicator of shorter overall and disease-free survival in a cohort of Chinese patients 30. The inverse situation we observed for Caucasian patients of the TCGA needs further evaluation.

In conclusion, EGFR is a relevant therapeutic target for mRCC in combination with anti-angiogenic treatment but only in the presence of a relevant mutation, different to those described in lung cancer. Association of first-generation immunotherapy with IFN should be revisited because of the associated debilitating side effects and new associations with immune checkpoint inhibitors may have a strong therapeutic impact.

## Supplementary Material

Supplementary figures and tables.

## Figures and Tables

**Table 1:**
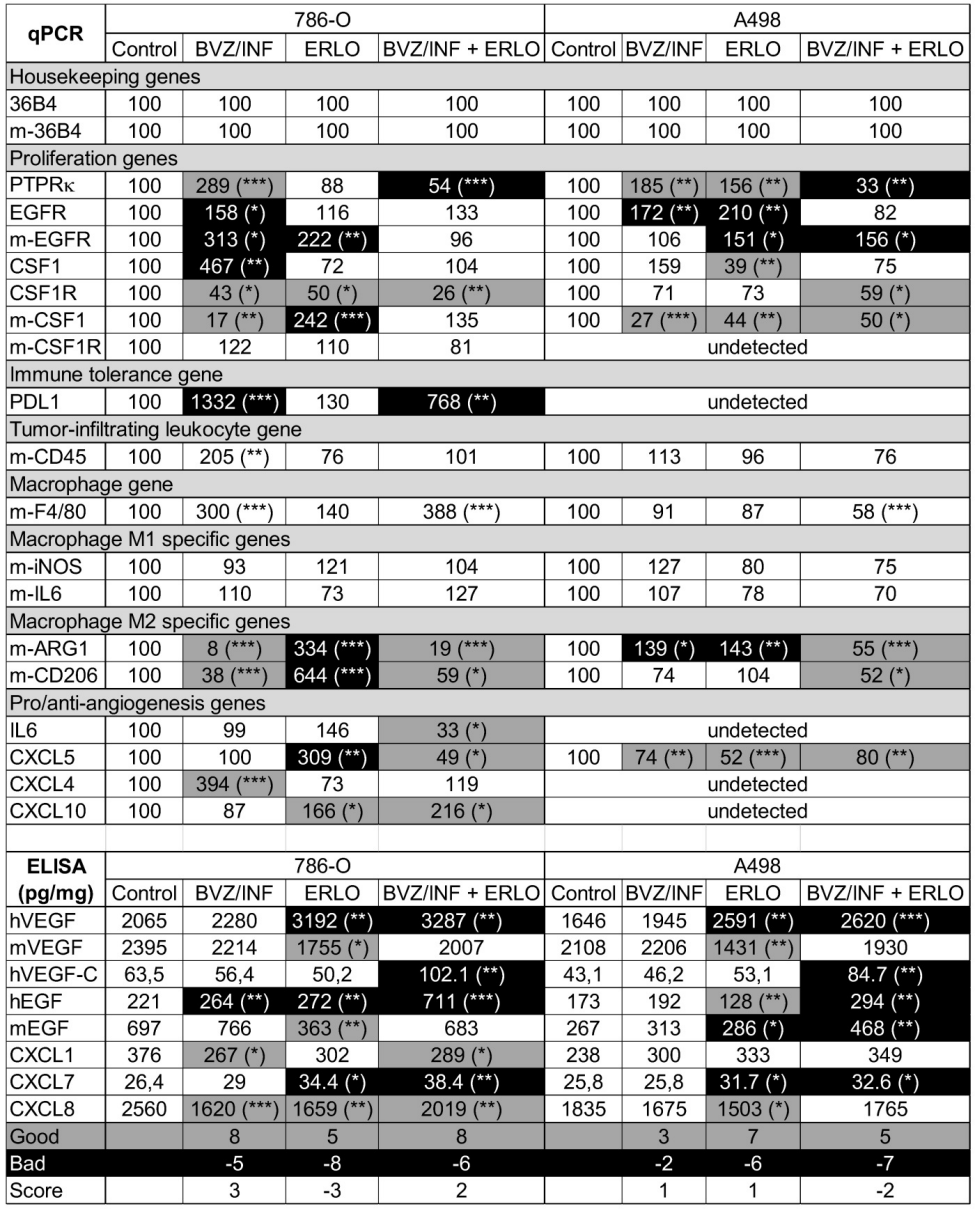
Analysis of pro-angiogenic/pro-lymphangiogenic/pro-inflammatory genes/proteins in tumors from mice treated with ERLO, BVZ/INF or BVZ/INF/ERLO.

The percentage expression of the different genes evaluated by qPCR and the amounts of cytokines detected by ELISA are shown. The indication “m” stands for mouse genes. If not indicated the genes are human ones. For the measured genes, the reference values (100) correspond to the content of a given gene in tumors of the placebo-treated mice. The amounts of cytokine in tumor extracts are given in picograms (pg) or nanograms (ng) per milligrams (mg) of total protein. The statistically significant differences are shown. * p < 0.05: ** p < 0.01:*** p < 0.001. A good prognostic marker is presented in black characters on a grey background; a poor prognostic marker is presented in white characters on a black background and markers with no significant modification are presented in black characters on a white background. The number of good or bad prognostic markers and the markers that are not influenced by a given treatment are shown. A score of +1 is given to a good prognostic marker whereas a score of -1 is given to a poor prognostic marker. The final score corresponds to the addition of good and poor prognostic markers. For 786-O cells, BVZ/INF and BVZ/INF/ERLO treatments gave positive scores (3 and 2 respectively) with the highest number of good prognostic indicators (8), whereas ERLO gave a negative score (-3) with the highest number of bad prognostic factors (-8). For A498 cells, BVZ/INF/ERLO treatment gave the worst score (-2) with the highest number of poor prognostic indicators (-7), whereas BVZ/INF and ERLO gave equivalent positive scores (1) with the highest number of good prognostic indicators for ERLO (7).

**Table 2:**
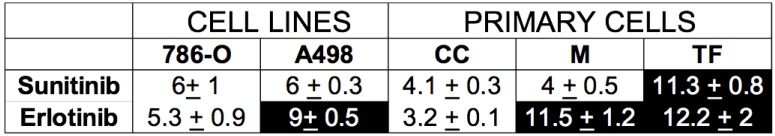
Sensitivity of the primary cells to the different treatments.

The IC50 for the different drugs ± SD is shown. 786-O cells are sensitive to sunitinib and erlotinib and serve as the reference. We considered the cells to be sensitive to a drug if the concentration giving 50% inhibition of cell proliferation (IC50) was lower than or equal to the IC50 in 786-O cells and was considered resistant if the IC50 was higher than for 786-O cells. CC, M and TF cells were derived from tumors of metastatic patients. When cells are sensitive to a given treatment, the value is presented on a white background but if cells are insensitive it is on a black background.
