# Supplementary material for: The combination of bevacizumab/Avastin and erlotinib/Tarceva is relevant for the treatment of metastatic renal cell carcinoma: the role of a synonymous mutation of the EGFR receptor
Source: Theranostics. 2020 Jan 1;10(3):1107–21. doi: 10.7150/thno.38346 (PMC6956821; doi:10.7150/thno.38346)
Supplement: Supplementary file 1 — Supplementary figures and tables. [file thnov10p1107s1.pdf]

Legends to supplementary figures

Figure S1. 786-O and A498 cells show different sensitivities to ERLO. Quantification of immuno-blots (three independent experiments), the representative images was shown on Fig. 1D. 786-O or A498 cells were treated with increasing concentrations of ERLO and were tested for the presence of total (EGFR/HSP90), the active form of the EGF receptor (pEGFR/EGFR), the active form of AKT (pAKT/HSP90) and the active form of ERK (pERK/ERK). The statistical significance for the different ERLO concentrations for a specific cell line is shown by * (* $p < 0.05$; ** $p < 0.01$). The statistical significance for the comparison of the two cell lines is shown by # (# $p < 0.05$; ## $p < 0.01$).

Figure S2. BVZ/IFN/ERLO on the vascular/lymphatic networks. The tumor vascular/lymphatic networks in each experimental group (control, B+I, E, B+I+E) were evaluated by CD31 immuno-staining and coverage of the vessels using an anti-〈SMA antibody as presented on Fig. 2. Vascular/lymphatic density (vessels/mm^2) and the number of vessels covered with 〈SMA labelled cells were determined using the Image J program. Quantification (means ± SD) resulted from analysis of four independent tumors and considered at least ten fields for each tumour. Statistically significant differences are indicated: * $p < 0.05$; ** $p < 0.01$; *** $p < 0.001$.

Figure S3. Differential inhibition of proliferation-mediating signaling pathways by ERLO in cells derived from experimental tumors. **(A)** Representative 786-O cells from the four experimental groups were tested for the presence of the total and active form of the EGF receptor (EGFR/pEGFR, see Fig. 3) and active forms of ERK (pERK) and AKT (pAKT). HSP90 is shown as a loading control. Quantification of the relative level of EGFR (EGFR/HSP90), pEGFR (pEGFR/EGFR), pERK (pERK/HSP90) and pAKT (pAKT/HSP90)

is shown (Fig. 3). The reference values (100%) correspond to the levels of the different parameters in cells of tumors derived from untreated mice in the absence of ERLO. **(B)** Equivalent experiments as described in **(A)** for the A498 model.

Figure S4. Parental 786-O cells proliferated faster than A498 parental cells. The proliferative capacity of parental 786-O and A498 cells was tested using the MTT assay. The results are presented as the mean percent increase ± s.d. Statistical differences are indicated; ** $p < 0.01$; *** $p < 0.001$.

Figure S5. RCC4, 786-O and A498 cells expressed different EGFR and EGFR-AS1 levels. **(A)** EGFR mRNA levels were evaluated by qPCR in RCC4, 786-O and A498 cells; **, $p<0.01$. **(B)** Representative immuno-blots showing the EGFR protein level in the different RCC cell lines after treatment in the absence or presence of increasing concentrations of ERLO. HSP90 is shown as a loading control. **(C)** Quantification in three independent experiments for EGFR levels in the different cell lines and in response to ERLO. ** $p < 0.01$; *** $p < 0.001$. **(D)** EGFR-AS1 mRNA levels were evaluated by qPCR in RCC4, 786-O and A498 cells; ***, $p<0.001$.

Figure S6. EGFR levels are correlated to RCC aggressiveness depending on the metastatic status. **(A)** Graph showing relative levels of EGFR transcript in RCC tumors of different stages versus normal tissue measured by RNA-seq data available from TCGA. p values are indicated. **(B)** The Kaplan–Meier analysis of overall survival (OS) of patients with M0 RCC from the TCGA data base. OS was calculated from patient subgroups with an optimized cut-off for EGFR levels. **(C)** The Kaplan–Meier analysis of overall survival of patients with M1 RCC from the TCGA data base. Overall survival was calculated from patient subgroups with an optimized

cut-off for EGFR levels. **(D)** The Kaplan–Meier analysis of disease-free survival (DFS) of patients with M0 RCC from the TCGA data base. DFS was calculated from patient subgroups with an optimized cut-off for EGFR levels. **(E)** The Kaplan–Meier analysis of progression-free survival (PFS) of patients with M1 RCC from the TCGA data base. PFS was calculated from patient subgroups with an optimized cut-off for EGFR levels.

Figure S7. EGFR-AS1 levels are correlated to RCC aggressiveness depending on the metastatic status. **(A)** Graph showing relative levels of EGFR-AS1 (1) transcript in RCC tumors of different stages versus normal tissue measured by RNA-seq data available from TCGA. p values are indicated. **(B)** The Kaplan–Meier analysis of overall survival (OS) of patients with M0 RCC from the TCGA data base. OS was calculated from patient subgroups with an optimized cut-off for EGFR-AS1 levels. **(C)** The Kaplan–Meier analysis of OS of patients with M1 RCC from the TCGA data base. OS was calculated from patient subgroups with an optimized cut-off for EGFR-AS1 levels. **(D)** The Kaplan–Meier analysis of disease-free survival (DFS) of patients with M0 RCC from the TCGA data base. DFS was calculated from patient subgroups with an optimized cut-off for EGFR-AS1 levels. **(E)** The Kaplan–Meier analysis of progression-free survival (PFS) of patients with M1 RCC from the TCGA data base. PFS was calculated from patient subgroups with an optimized cut-off for EGFR-AS1 levels.

Reference

1. Tan DSW, Chong FT, Leong HS, Toh SY, Lau DP, Kwang XL, *et al.* Long noncoding RNA EGFR-AS1 mediates epidermal growth factor receptor addiction and modulates treatment response in squamous cell carcinoma. Nat Med **2017**;23:1167-75

	FORWARD	REVERSE
**Housekeeping genes**		
36B4	CAGATTGGCTACCCAACTGTT	GGCCAGGACTCGTTTGTACC
m-36B4	AGATTCGGGATATGCTGTTGGC	TCGGGTCCTAGACCAGTGTTC
GAPDH	TGC ACC ACC AAC TGC TTA GC	GGC ATG GAC TGT GGTCAT GAG
**Pro/Anti-angiogenesis genes**		
m-CD31	ACGCTGGTGCTCTATGCAAG	TCAGTTGCTGCCCATTCATCA
m-NG2	ACTAACCCATGCACTACATCAAG	ACTTTTCCAGACAGAGAGCCTT
m-αSMA	GTC CCA GAC ATC AGG GAG TAA	TCG GAT ACT TCA GCG TCA GGA
IL6	CCTGAACCTTCCAAAGATGGC	TTCACCAGGCAAGTCTCCTCA
CXCL5	AGCTGCGTTGCGTTTGTTTAC	TGGCGAACACTTGCAGATTAC
CXCL4	AGCCACGCTGAAGAATGGAA	CACACACGTAGGCAGCTAGT
CXCL10	GTGGCATTCAAGGAGTACCTC	TGATGGCCTTCGATTCTGGATT
**Lymphangiogenesis genes**		
mLYVE	CAG CACACTAGCCTGGTGTTA	CGCCCATGATTCTGCATGTAGA
m-VEGF-C	CTCTGTGGGACCACATGGTAA	TCCTCTCCCGCAGTAATCCA
**Proliferation genes**		
PTPRk	AATGCTCCTCCTCAGCTTCTTGGT	AGGACCATCGCCATTGATCGAGTT
EGFR	ATGCGACCCTCCGGGACGGC	CAAACGGTCACCCC TTTCTTTTCC
EGF	TGTCCACGCAATGTGTCTGAA	CATTATCGGGTGAGGAACAACC
m-EGFR	GCCATCTGGGCCAAAGATACC	GTCTTCGCATGAATAGGCCAAT
m-EGF	AGCATCTCTCGGATTGACCCA	CCTGTCCCGTTAAGGAAAACTCT
CSF1	TGGCGAGCAGGAGTATCAC	AGGTCTCCATCTGACTGTCAAT
m-CSF1	ATGAGCAGGAGTATTGCCAAGG	TCCATTCCCAATCATGTGGCTA
m-CSF1R	TGTCATCGAGCCTAGTGGC	CGGGAGATTCAGGGTCCAAG
**Immune tolerance genes**		
PDL1	TGGCATTTGCTGAACGCATTT	TGCAGCCAGGTCTAATTGTTTT
**Macrophage M1 genes**		
miNOS	TCACCTTCGAGGGCAGCCGA	TCCGTGGCAAAGCGAGCCAG
mIL6	ATCCAGTTGCCTTCTTGGGACTGA	TTGGATGGTCTTGGTCCTTAGCCA
**Macrophage M2 genes**		
mARG1	GATTATCGGAGCGCCTTTCT	CCACACTGACTCTTCCATTCTT
mCD206	CTGCAGATGGGTGGGTTATT	GGCATTGATGCTGCTGTTATG

Supplementary Table S1. List of oligo-nucleotides used in qPCR experiments

	ERLO (μM)	EGFR/HSP90	pEGFR/EGFR	pAKT/HSP90	pERK/ERK
786-O	0	100	100	100	100
	1	120	17 **	77 *	30 **
	10	140	1 **	53 *	30 **
A498	0	247 ##	1 #	14 #	279 ##
	1	184	5	23	198 *
	10	240	1	23	151 *

Supplementary Figure S1: Grépin *et al*

A

786-O	Control	B + I	E	B + I + E
CD31	10	6.6	6 (*)	4.75 (**)
CD31 + αSMA	0.75	3 (***)	2.25 (***)	1.25 (***)
LYVE1	1.2	4 (***)	2.3 (**)	1.6 (*)

B

A498	Control	B + I	E	B + I + E
CD31	24	25	18.8	15.6 (**)
CD31 + αSMA	9.4	18.8 (***)	14.4 (***)	8.2 (*)
LYVE1	4.7	7.5 (**)	3.5 (***)	3.3 (***)

Supplementary Figure S2: Grépin *et al*

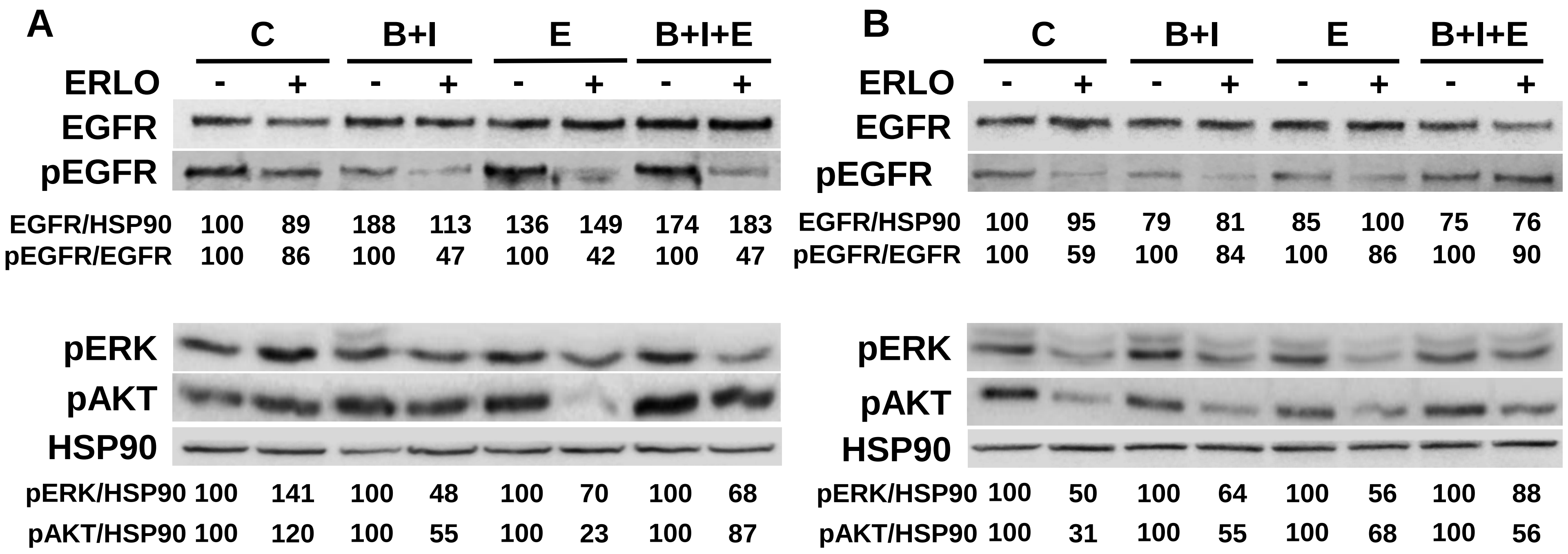

Supplementary Figure S3: Grépin *et al*

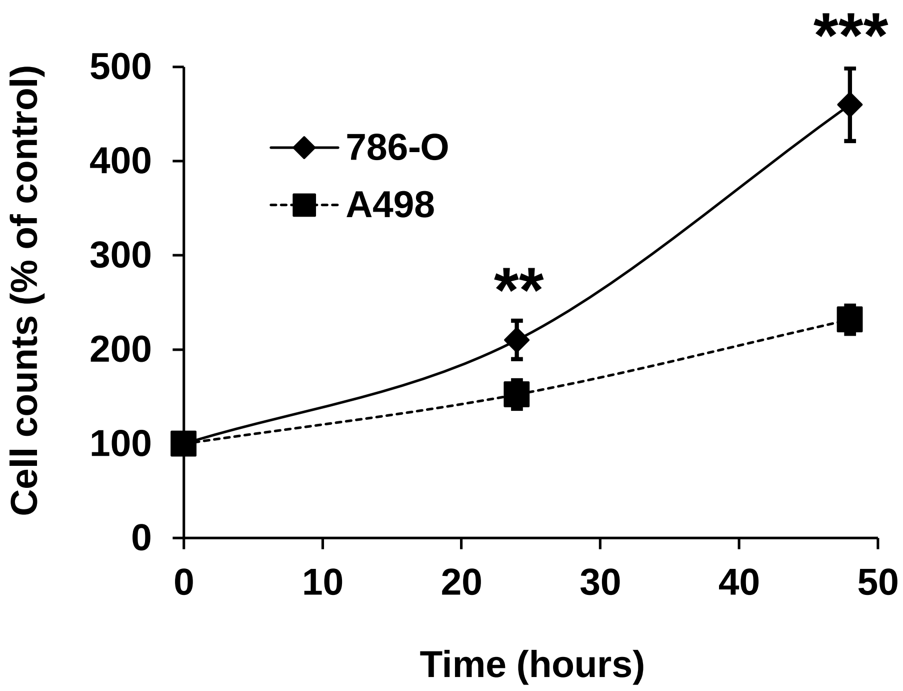

Supplementary Figure S4: Grépin *et al*

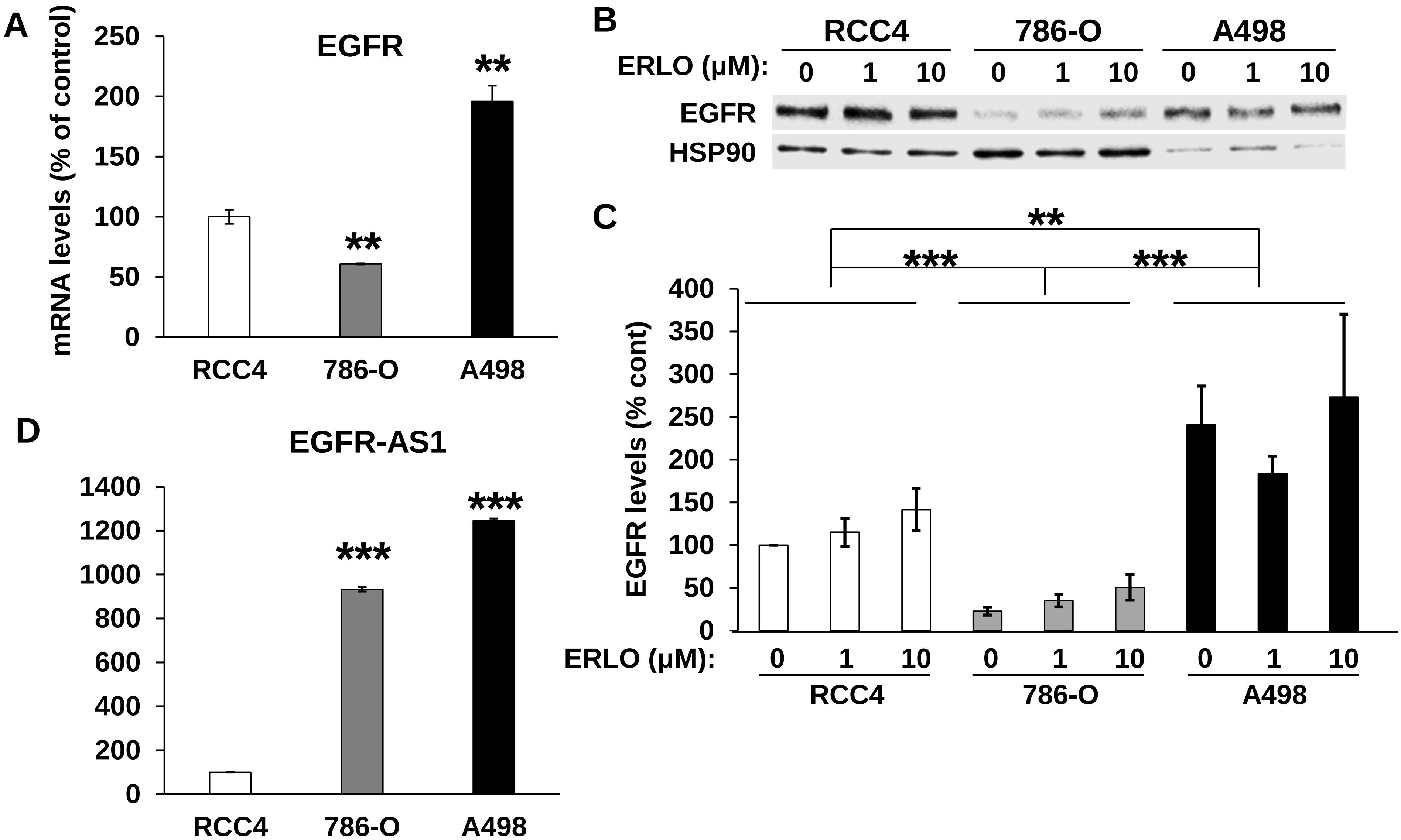

Supplementary Figure S5: Grépin *et al*

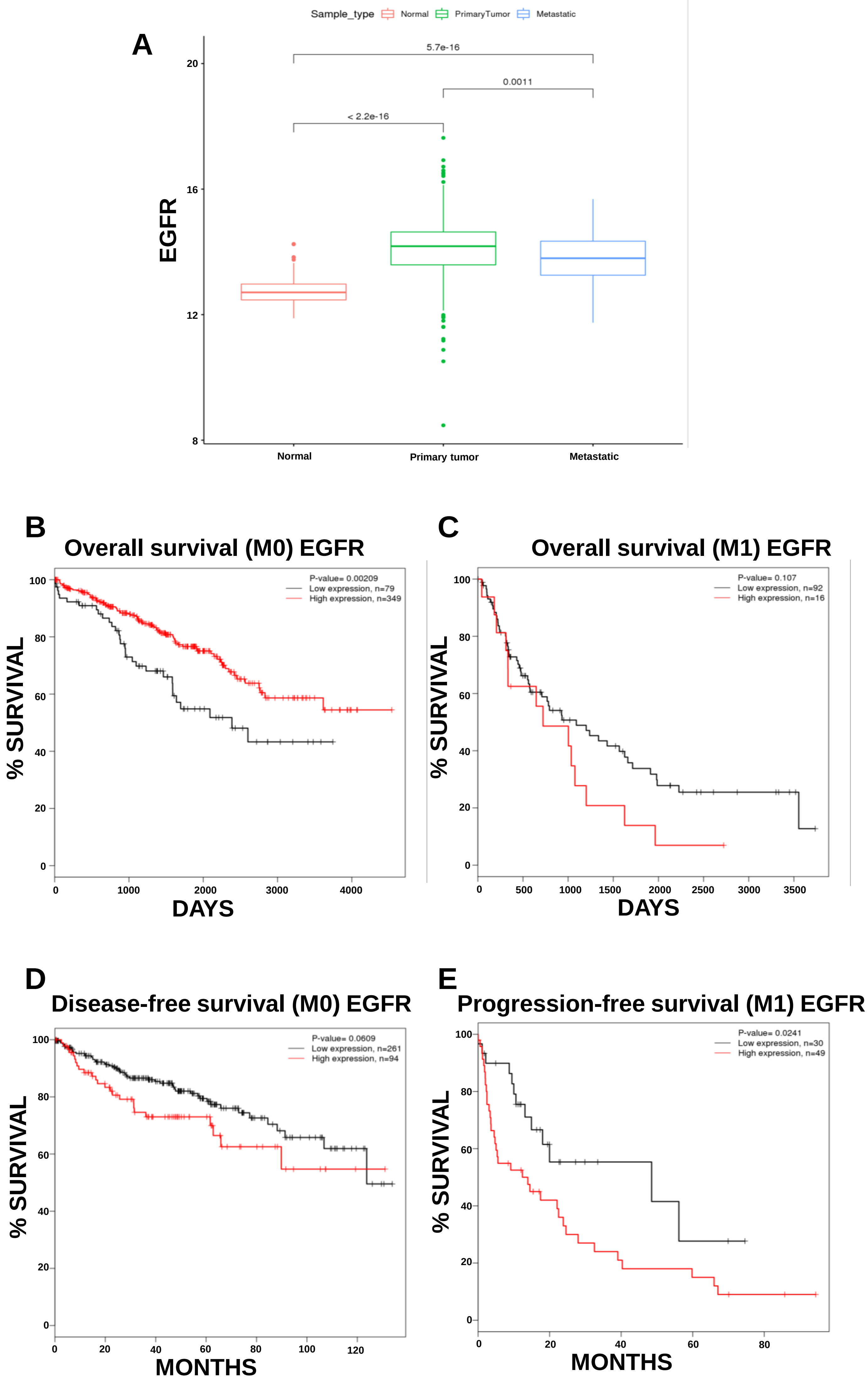

Supplementary Figure S6: Grépin *et al*

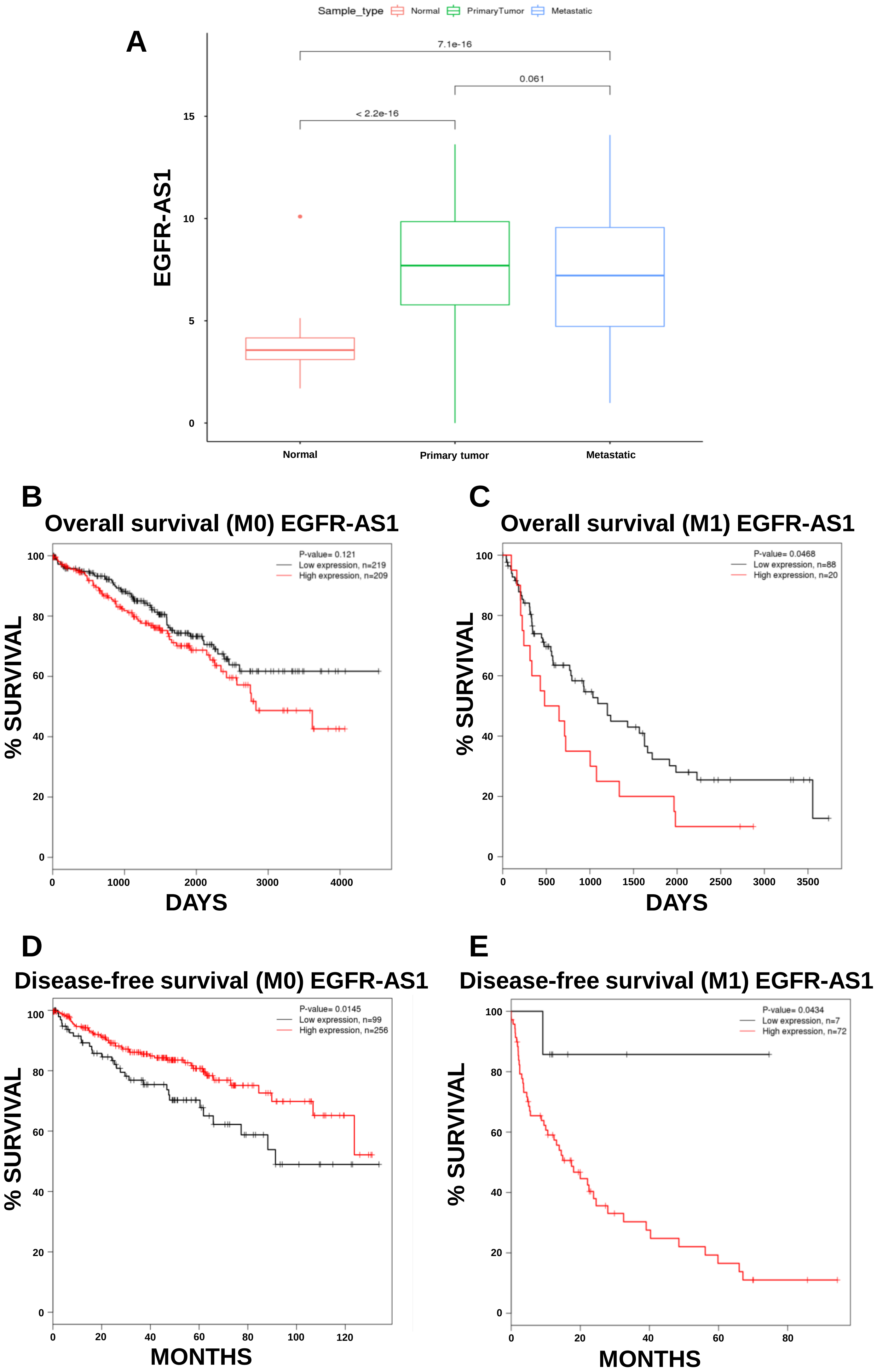

Supplementary Figure S7: Grépin *et al*
